# Molecular Analysis of Endocrine Disruption in Hornyhead Turbot at Wastewater Outfalls in Southern California Using a Second Generation Multi-Species Microarray

**DOI:** 10.1371/journal.pone.0075553

**Published:** 2013-09-25

**Authors:** Michael E. Baker, Doris E. Vidal-Dorsch, Cataldo Ribecco, L. James Sprague, Mila Angert, Narimene Lekmine, Colleen Ludka, Andrea Martella, Eugenia Ricciardelli, Steven M. Bay, Joseph R. Gully, Kevin M. Kelley, Daniel Schlenk, Oliana Carnevali, Roman Šášik, Gary Hardiman

**Affiliations:** 1 Department of Medicine, University of California San Diego, La Jolla, California, United States of America; 2 Southern California Coastal Water Research Project, Costa Mesa, California, United States of America; 3 Department of Life and Environmental Sciences, Università Politecnica delle Marche, Ancona, Italy; 4 BIOGEM, School of Medicine, University of California San Diego, La Jolla, California, United States of America; 5 Los Angeles County Sanitation Districts, Whittier, California, United States of America; 6 Environmental Endocrinology Laboratory, California State University, Long Beach, California, United States of America; 7 Department of Environmental Sciences, University of California Riverside, Riverside, California, United States of America; Glasgow Caledonian University, United Kingdom

## Abstract

Sentinel fish hornyhead turbot ( *Pleuronichthys* *verticalis* ) captured near wastewater outfalls are used for monitoring exposure to industrial and agricultural chemicals of ~ 20 million people living in coastal Southern California. Although analyses of hormones in blood and organ morphology and histology are useful for assessing contaminant exposure, there is a need for quantitative and sensitive molecular measurements, since contaminants of emerging concern are known to produce subtle effects. We developed a second generation multi-species microarray with expanded content and sensitivity to investigate endocrine disruption in turbot captured near wastewater outfalls in San Diego, Orange County and Los Angeles California. Analysis of expression of genes involved in hormone [e.g., estrogen, androgen, thyroid] responses and xenobiotic metabolism in turbot livers was correlated with a series of phenotypic end points. Molecular analyses of turbot livers uncovered altered expression of vitellogenin and zona pellucida protein, indicating exposure to one or more estrogenic chemicals, as well as, alterations in cytochrome P450 (CYP) 1A, CYP3A and glutathione S-transferase-α indicating induction of the detoxification response. Molecular responses indicative of exposure to endocrine disruptors were observed in field-caught hornyhead turbot captured in Southern California demonstrating the utility of molecular methods for monitoring environmental chemicals in wastewater outfalls. Moreover, this approach can be adapted to monitor other sites for contaminants of emerging concern in other fish species for which there are few available gene sequences.

## Introduction

Many synthetic chemicals alter endocrine responses in humans and wildlife causing adverse effects on development, reproduction and the incidence of diseases such as diabetes and hypertension [1-8]. These chemicals are called endocrine disruptors, and they include plasticizers, such as phthalates and alkylphenols, pesticides, fungicides, detergents, dioxin, polychlorinated biphenyls and pharmaceuticals, such as the synthetic estrogen 17α-ethinylestradiol. These and other chemicals from municipal wastewater treatment and industrial sources are discharged into rivers, lakes and the ocean, where they accumulate in aquatic species [9]. Humans and wildlife may be exposed to these compounds through fish and shellfish consumption. In addition, humans are exposed to endocrine disruptors via polluted drinking water. As a result, there is much concern about the effects of elevated concentrations of xenobiotics in the environment on human health because exposure to endocrine disruptors may lead to premature puberty in females [5,10,11], and decreased reproductive ability in men [12,13].

There are over 20 million people living within 50 km of the coastline in the region in southern California, encompassing Los Angeles, Orange and San Diego Counties. This coastal region contains extensive industrial, commercial, residential and agricultural activities, which release a wide variety of chemicals into the environment. Servicing these urban centers are wastewater treatment plant (WWTP) facilities, which release more than 1 billion gallons (4 billion liters) of treated wastewater each day into the coastal marine environment. In addition to inputs of municipal wastewaters, there are additional sources of wastewater, pesticides and other contaminants, coming from urban runoff, storm water drainage, ports and harbors, agriculture and legacy contamination (e.g., DDT, PCB) among others.

The growing world-wide evidence that exposure to low levels of chemicals can lead to disruption of hormone-mediated responses in fish [6,14-16], which to visual inspection seem healthy, motivated these agencies to collaborate with the Southern California Coastal Water Research Project (SCCWRP) and university research groups in Long Beach, Riverside and San Diego to develop a microarray tool as a sensitive and quantitative measure of endocrine disruption in hornyhead turbot, a sentinel species collected from southern California waters. A species that has been relatively well studied in this area over the past ten years is the hornyhead turbot ( *Pleuronichthys* *verticalis* ). An obstacle to this goal was that there are few gene sequences for hornyhead turbot in GenBank. Although DNA sequence is available from a related demersal species ( *Scophthalmus* *maximus* ) [17,18] native to the North Atlantic and Baltic and Mediterranean Seas, at present the genome of the hornyhead turbot remains to be sequenced and consequently sequences for turbot vitellogenin (*Vtg*), cytochrome P4503A (*CYP3A*) and other genes that are biomarkers for endocrine disruption are not available. To overcome this obstacle, we previously developed a multi-species microarray containing key genes such as vitellogenin [Vtg], estrogen receptor [ER], glucocorticoid receptor [GR], thyroid hormone receptor [TR], *CYP1A* and *CYP3A*. This prototype microarray and a corresponding Q-PCR gene panel successfully measured expression of various genes involved in the responses to steroids, thyroid hormone, retinoids and growth factors in livers of turbot collected off of Los Angeles and Orange, County, California [19]. In this earlier study, we tested the multi-species applicability of this tool using microarray measurements of gene expression in zebrafish, which are phylogenetically distant from turbot. The effects of estradiol and the aquatic pollutant nonylphenol on liver gene expression in male zebrafish were investigated with this microarray, demonstrating its applicability for measuring endocrine responses in turbot and other fish [19], Thus, we call this a multi-species microarray both because the probes in its design come from different fish species and because it can potentially be used with diverse fish species.

Building on this initial study, a “second generation” microarray was constructed that includes additional gene targets such as zona pellucida protein (also known as choriogenin), glutathione S-transferase-α, metallothionein and heat shock protein 90, which are diagnostics for endocrine disruption and the presence of metals and stress responses. We used this optimized tool in a new and more ambitious study to investigate endocrine disruption in hornyhead turbot (a species which remains to have its genome sequenced) collected near outfalls for municipal wastewater for Los Angeles County Sanitation Districts (LACSD), Orange County Sanitation District (OCSD), City of Los Angeles Environmental Monitoring Division (CLAEMD), and City of San Diego Metropolitan Wastewater Department (MWWD). As reported here, this multi-species microarray was able to characterize changes in gene expression in hornyhead turbot collected from wastewater outfalls for municipal wastewater in coastal waters off of southern California. We correlated gene expression data from microarray analysis and Q-PCR with a series of phenotypic endpoints in fish from impacted sites. This validates our multi-species approach as a practical diagnostic screening tool to monitor responses to contaminants in hornyhead turbot collected from different sites, despite the genetic heterogeneity in wild fish, which would be expected to diminish the resolution of the microarray output. We also note that the multi-species microarray can be potentially adapted to monitoring endocrine disruption near other population centers in other fish species, for which there are few available gene sequences.

## Results

### Improved performance characteristics of the second generation multi-species endocrine microarray

**Figure 1:**
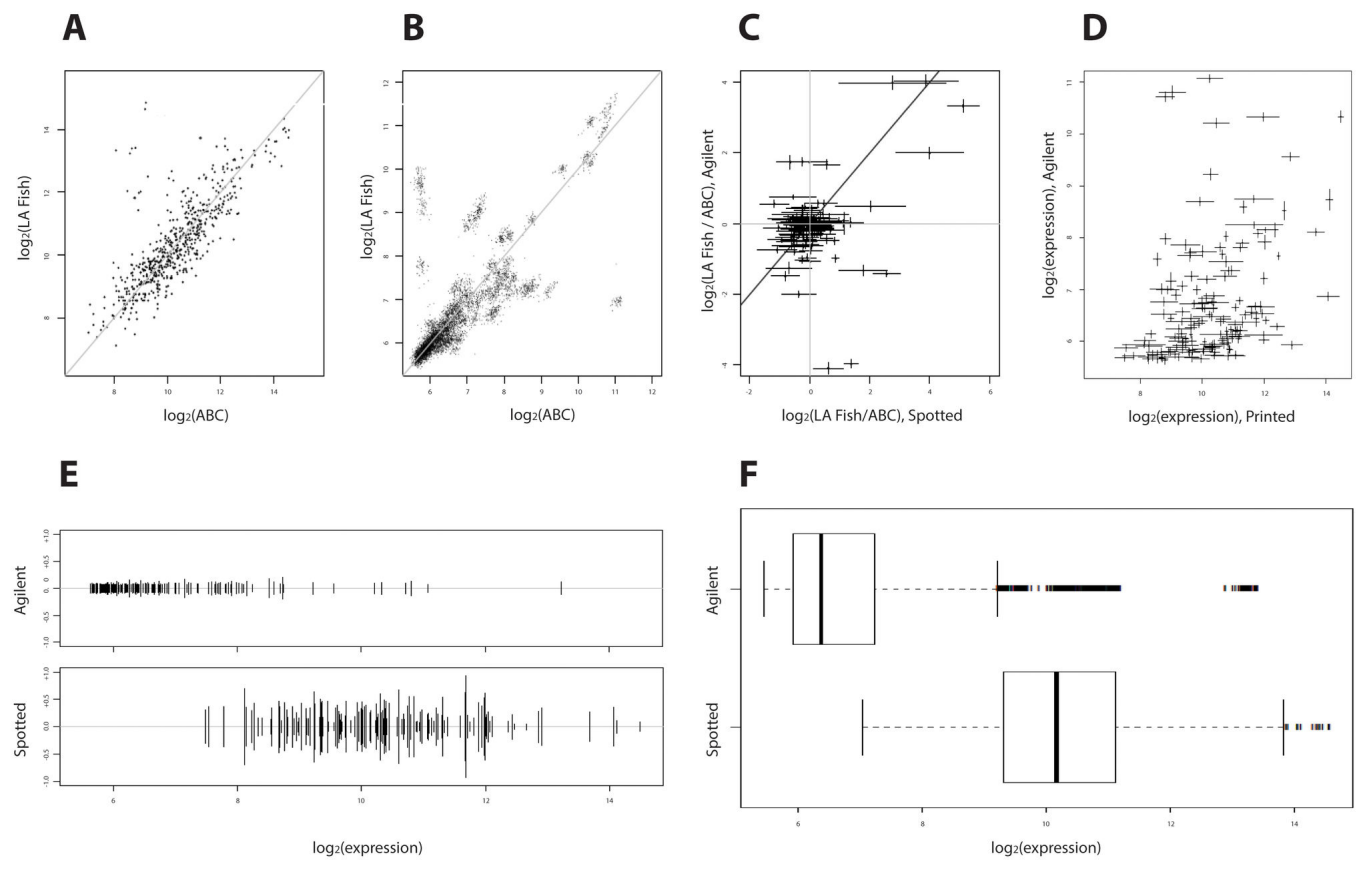
Quality control specifications for the second generation multi-species endocrine microarray. Comparison of the performance characteristics of on both prototype (spotted oligonucleotide) and second generation (Agilent ink jet oligonucleotide) endocrine multi-species microarray platforms; Panels A and B: scatter plots of log_2_ signal intensities of an individual fish from the Los Angeles Sanitation District versus the control fish pool. Panels A and B depict prototype and second generation platforms respectively. Panel C; direct comparison of platform sensitivity defined as log_2_ ratio between treatment and ABC control. Panel D: Analysis of signal concordance. Panel E: Analysis of variance with both platforms. To measure internal consistency (variance of internal replicates), log_2_ transformed expression value was plotted on the x-axis. Standard deviation is plotted on the y-axis, with a range of plus or minus one standard deviation from the mean expression value. The y-axis is a measure of the deviation of individual replicate probes stray from the mean value. The second generation platform has smaller variance of the internal replicates. Probe variance on both platforms is independent of the signal. Panel F: Comparison of the probe signal intensity distribution (plotted as log_2_ transformed) for 149 probes that were present on both platforms.

To construct the multi-species microarray, we queried GenBank for sequences in a variety of fish to uncover regions of sequence conservation that would permit design of a microarray suitable for detecting altered gene expression in multiple fish species (Figure S1). The improved or second generation multi-species array was fabricated by Agilent ink jet printing and contained expanded probe content (Table S1). We utilized a series of quality control metrics to validate the second generation microarray with turbot (Agilent ink jet oligonucleotide) and compared its performance to the prototype (spotted oligonucleotide) we described previously (Figure 1). Scatter plots of the log_2_ signal intensities of an individual impacted fish from the Los Angeles Sanitation District versus the control fish pool are shown for both platforms (Figure 1A and B). Data from all probes present on both platforms were plotted for comparative purposes. Replicate probes clustered together better in the second generation array (Figure 1B) indicating better signal to noise ratios. A direct comparison of the sensitivity of both platforms, defined as the log_2_ ratio between the impacted and control fish pool is presented in Figure 1C. Data from the prototype and second generation microarray are plotted on the x and y axes respectively. Data points should line up along the shaded diagonal if both platforms performed equally. The large number of points centered on the origin indicates that 2-fold and higher changes when measured by the spotted oligonucleotide array are robust. Data points fall in the first and third quadrants, indicating that the platforms are in agreement when fold changes are high (e.g. ~2^4^). However data points also fall in the fourth quadrant which correspond to transcripts that are downregulated as measured by the second generation platform but which were not detected with the prototype array (Figure 1C). This correlates with the distribution of log_2_ intensities in Figure 1A, which is broad and symmetric, with just a few probes clustering at the far right tail. The noise is such with the spotted oligonucleotide array that non-expressed genes appear in the center of the distribution. This suggests that when a gene’s expression is down regulated, after inclusion of this noise, it will still appear in the center of the distribution and appear as though there has been no change in gene expression. We analyzed signal concordance between the two platforms (Figure 1D). Each point corresponds to the same probe sets, the crosshairs are ± one standard deviation either way as determined from internal replicates. This plot measures concordance of the two platforms in the general sense that if, for two genes X and Y, we have signal strength S(X) > S(Y) on one platform, we would expect this relationship to be true for the other platform as well because the probe sequences are essentially the same. However as the probe sequences are not exactly the same, because the second generation Agilent array contains truncated probes (60-mers) compared to the prototype (65-mers), thus we do not expect a perfectly straight line. The curvature observed in the line plus some deviations either way fall within performance expectations for the platforms. To measure the internal consistency of probe performance between platforms, the log_2_ expression value was plotted on the x-axis and standard deviation plotted on the y-axis (Figure 1E). The y-axis is therefore a measure of the deviation of individual replicate probes from the mean value. Probe variance on both platforms was independent of the signal strength. However the second generation array exhibited a smaller variance of the internal replicates when compared to the prototype spotted oligonucleotide platform. As a final measure of both platforms we examined the probe signal intensity distribution for 149 probes that were present on both the prototype and second generation microarrays (Figure 1F). Each 65-mer probe was replicated 4 times on the spotted oligonucleotide array and the corresponding 60-mer 46 times on the Agilent array. The distributions of log_2_ transformed raw signal intensities from both platforms were plotted. The prototype exhibited higher overall signal. The second generation array data followed an asymmetric distribution, which is preferable as a peak at low intensity. A long tail to the right is indicative that expressed genes are sufficiently resolved from those which are not expressed. Although the data from the prototype array also followed an asymmetric distribution, the greater noise with this platform causes non-expressed genes to mix with expressed ones, potentially blurring the sensitivity of the platform.

### Differential expression and signal intensity measurements using the second generation multi-species microarray

After the initial optimization experiments and determination that the second generation array was superior, we assessed hornyhead turbots sampled from five field sites in southern California for exposure to endocrine disruptors. The sites in California included sanitation districts in Los Angeles City (three fish; LA 27, LA 41, LA 46) and Los Angeles County (two fish; PV-25, PV-15), Orange, County (seven fish; OC 13, OC 29, OC 7, OC 37, OC 20, OC 43, OC 34), San Diego (three fish; SD 07, SD 29, SD 46), all of which are considered impacted and a reference site Dana Point (seven fish DP 03, DP 37, DP 08, DP 44, DP 29, DP 32, DP 39), an area relatively distant from the main municipal wastewater outflows. Three control fish were obtained from a separate monitoring station in Dana Point, an area relatively distant from the main municipal wastewater outflows. After collection, the control fish were taken to a laboratory in the Cabrillo Marine Aquarium (San Pedro, CA) where they were placed in tanks with clean seawater which was renewed with a flow-through system for four weeks. The rationale for use of these fish as a reference for studying environmental endocrine disruption was they exhibited greater homogeneity in gene expression profiles than individual wild fish sampled from different exposed sites. The fish from the sites under study reflected the natural variance in gene expression profiles typical of outbred populations.

**Figure 2:**
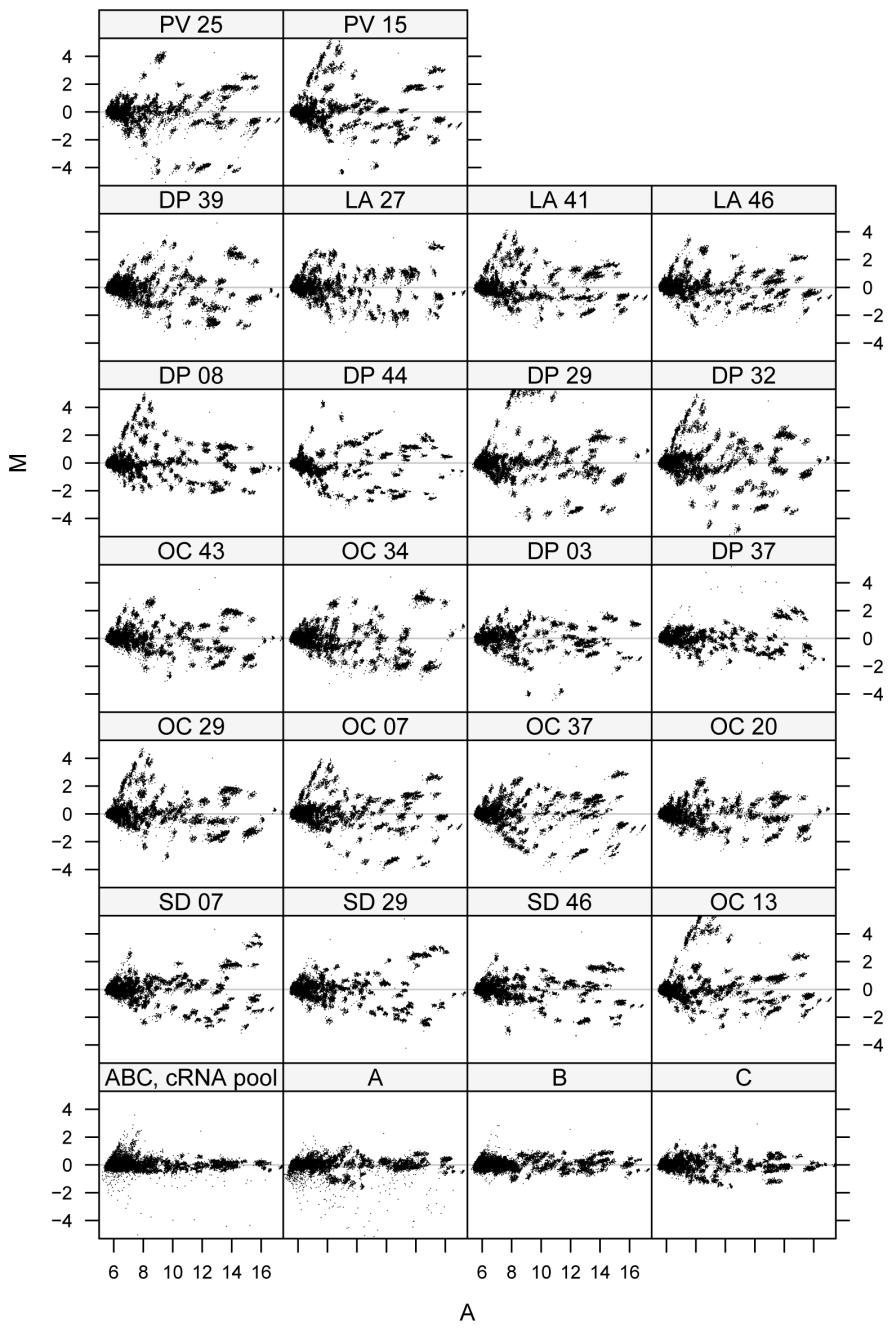
Differential expression and signal intensity measurements for impacted turbot livers. A: Gene expression changes were investigated in male turbot liver collected from exposed fish sampled from sanitation districts in San Diego, Orange County, Dana Point, Los Angeles City and Los Angeles County in California that are considered impacted. Control fish were obtained from a separate monitoring station in Dana Point, a relatively non-impacted area and maintained in a clean-water laboratory for four weeks. The control reference sample consisted of a pool of labeled cRNA from three control fish, designated *A*, *B*, and *C*. M (Y-axis) is a measure of differential gene expression (log2 (exposed /control) in the samples in the first three panel rows of plots or absence of significant differential gene expression in the self-self plots (log_2_ (control / control intensity) in the bottom panel row of plots. A (X-axis) is a measure of signal intensity (0.5 log_2_ exposed intensity + 0.5 log_2_ control intensity) in the first three panel rows of plots or (0.5 log_2_ control intensity + 0.5 log_2_ control intensity) in the bottom panel row of plots. Careful analysis of the heavy tail of outliers for the individual fish *A* (second panel from right, bottom row), revealed these data points in the tail are not real and are derived from an array artifact.

We examined hepatic RNA from male fish collected from the field using the multi-species microarray. The characteristics of male hornyhead turbot sampled and the chemical analysis of sediments from the sites where the fish were captured are provided in Tables 1 and 2 respectively. M-A scatter plots were used to examine differences in mRNA expression levels between the indicated fish samples and a pooled reference sample, derived from three individual control fish samples A, B, and C (Figure 2). This same pooled reference sample (A + B + C) was used for every comparison. Differences in gene expression in hornyhead turbot liver relative to the reference fish were determined using a threshold of log_2_ intensity ratio > 2. The bottom row of plots in Figure 2 was used to compare the pooled reference (A + B + C) versus the individual reference fish reference samples, A, B, and C respectively that comprised the reference. This revealed that the order in which the mixing and labeling of control RNA was carried out had minimal effects on the overall performance of the pooled reference, as no differential gene expression was detected. The MA plots revealed differential gene expression profiles between the pooled reference and hornyhead turbot sampled from the five field sites in southern California. Although we noted a heavy tail of outliers for the reference fish A versus the pooled reference (second panel, bottom row), further analysis revealed that this resulted from an array hybridization artifact. We selected just the probes that are present in this tail (M < -1.5), and plotted their row and column coordinates in a 2D plot (Figure S2). The probes were concentrated along a line, evidence that this represents a microarray artifact, and that caution is warranted when performing differential expression measurements using microarrays.

**Table 1:** Characteristics of male hornyhead turbot sampled.

**Station**	**Sample**	**Date**	**VTG (ng/ug protein**)	**E2 (pg/ml**)	**Cort (ng/ml**)	**T4 (ng/ml**)	**T (ng/ml**)	**IGF-I (ng/ml**)	**Calc Age (y**)	**SL (cm**)
LA	27	6/13/2006	0.05	417.6	2.72	28.95	1.47	29.39	8	16
LA	41	6/20/2006	0.06	590.3	54.4	31.93	1.72	21.76	8	16
LA	46	6/20/2006	0.12	261.3	95.6	37.59	0.96	28.7	9	17.1
PV	15	6/7/2006	0.09	716.8	14.33	27.55	1.22	32.52	8	16.5
PV	25	6/7/2006	0.06	569.2	5.25	14.23	0.73	36.93	8	16.7
OC	7	5/23/2006	0.07	116.2	87.02	27.09	1.35	20.34	7	15
OC	13	5/23/2006	0.03	156.1	30.22	32.34	1.97	22.39	7	15.5
OC	20	5/23/2006	0.04	200.5	79.2	33.61	1.58	23.61	6	14
OC	29	5/25/2006	0.04	151.6	46.99	3.89	1.21	25.58	7	15
OC	34	5/25/2006	0.09	281.3	40.37	34.29	1.69	20.67	6	13
OC	37	5/25/2006	0.37	219.2	41.91	37.04	1.43	23.89	7	15.4
OC	43	5/25/2006	0.15	274.8	26.5	28.09	1.6	17.87	7	14.8
DP	3	5/23/2006	0.05	1512.6	143.64	53.94	1.3	8.46	7	15.3
DP	8	5/23/2006	0.07	1102	49.43	28.79	2.12	15.24	7	14.9
DP	29	5/23/2006	0.01	321.1	23.42	41.32	0.74	25.74	8	16
DP	32	5/23/2006	0.03	1209.3	26.32	36.95	1.13	22.91	6	13.7
DP	37	6/30/2006	0.06	493.7	31.64	105.4	3.56	34.01	7	14.4
DP	39	6/30/2006	0.03	268.1	39.47	55	3.58	25.43	6	13.2
DP	44	6/30/2006	0.01	611.7	1.12	46.59	2.37	27.32	8	16
SD	7	6/22/2006	0.08	1988.4	56.77	16.46	1.74	24.89	6	12.3
SD	29	6/22/2006	0.2	377.9	7.71	47.49	1.99	25.93	6	12.2
SD	46	6/23/2006	0.06	473.7	86.95	47.37	0.84	32.71	6	11.4

All fish analyzed were male. Individuals with morphological abnormalities induced by EDs were chosen for microarray experiments. Concentrations of vitellogenin (VTG), estradiol (E2), cortisol (Cort), thyroxine (T4), testosterone (T), and insulin-like growth factor (IGF-1) are listed with, calculated age (Calc Age), standard length (SL) and weight (Wt) values. The sampling date and Station ID are indicated. Table modified from [46].

**Table 2:** Chemical analysis of sediments from the sites where the fish were captured.

**Station ID**	**Chemical**	**Result**
Dana Point [DP]	4,4’-DDT	Below Detection Limit
Dana Point [DP]	4-nonylphenol	10 µg/kg
Dana Point [DP]	PBDEs	Below Detection Limit
Dana Point [DP]	PCBs	Below Detection Limit
Orange County [OC]	4,4’-DDT	Below Detection Limit
Orange County [OC]	4-nonylphenol	380 µg/kg
Orange County [OC]	PBDEs	38 µg/kg
Orange County [OC]	PCBs	1.2 µg/kg
San Diego [SD]	4,4’-DDT	Below Detection Limit
San Diego [SD]	4-nonylphenol	10 µg/kg
Los Angeles County [PV]	PBDEs	Below Detection Limit
Los Angeles County [PV]	PCBs	Below Detection Limit
Los Angeles County [PV]	4,4’-DDT	50 µg/kg
Los Angeles County [PV]	4-nonylphenol	110 µg/kg
Los Angeles County [PV]	PBDEs	42 µg/kg
Los Angeles County [PV]	PCBs	98.4 µg/kg
Los Angeles City [LA]	4,4’-DDT	Below Detection Limit
Los Angeles City [LA]	4-nonylphenol	30 µg/kg
Los Angeles City [LA]	PBDEs	39 µg/kg
Los Angeles City [LA]	PCBs	34 µg/kg

DDT = dichlorodiphenyltrichloroethane

PCB = polychlorinated biphenyl

PBDE = Polybrominated diphenyl ether

In addition to MA plots (Figure 2) Box plots were employed to examine more closely the distribution of feature intensities among fish from the field sites (Figure S3). The x-axis on each box plot represents the log intensity ratio data (M) corresponding to the three pooled reference fish samples (A + B + C); the three individual control samples A, B, and C respectively, and the fish sampled from each of the five field stations in southern California described above. M measured differential gene expression [log_2_ (sample/pooled reference ABC)] for all these samples, and only data from genes considered detected were plotted. The individual control fish A, B, and C exhibited a much smaller inter-quartile range (grey rectangles) than any of the fish sampled from field sites (white rectangles) (Figure S3). The fish from the five field sites exhibited wider distributions in differentially expressed genes and outliers relative to the reference fish, indicating that a large number of genes are differentially regulated in response to the environment in which they were sampled. The presence of a large number of notches, representing outliers, in the individual sample A were the result of the hybridization artifact described above, and these probes were removed from any subsequent analysis.

### Gene expression patterns in male turbot from southern California coastal regions

We carried out feature level analysis of the top ranked differentially regulated probes on the multi-species array and a heat map was generated of selected probes representing 21 genes that were either strongly down- or up-regulated in fish collected from the five field stations relative to the pooled laboratory reference (Figure S4). The fold changes were determined from log_2_ ratios between the probe signal of each individual fish and that of the pooled control sample. There were 326 unique probes present on the array, each of which was replicated in 46 distinct spatial locations throughout the array. The log_2_ ratio value was calculated for each probe as the median of the 46 replicate log_2_ intensity ratios. The 326 unique probes were subsequently sorted by their importance in descending order of the sum-squared statistic (i.e., sum of squares of log_2_ ratios across all fish) as described previously [19,20]. The rationale behind this approach was that it provided a measure of change in expression values for any one or all exposed fish. In this manner the sum-squared statistic measured the amount of variance across any and all exposed sites, i.e. a transcript with altered expression in one fish, from location A would be selected along with another transcript with altered expression in a separate fish, from location B. The top ranked 100 probes were arbitrarily selected and included in the heat map. Since the majority of genes were represented on the multi-species microarray by sequences derived from several fish species, we further consolidated probes from the same gene, albeit different species, into a color strip of the same width. In the heat map the *ER* and *Vtg* strips contains probe data from two and five different species respectively. In this way, we were able to reduce the top 100 probes to 21 differentially regulated genes. In the majority of cases, the inter-species data consistency was highly consistent validating the multi-species approach.

**Figure 3:**
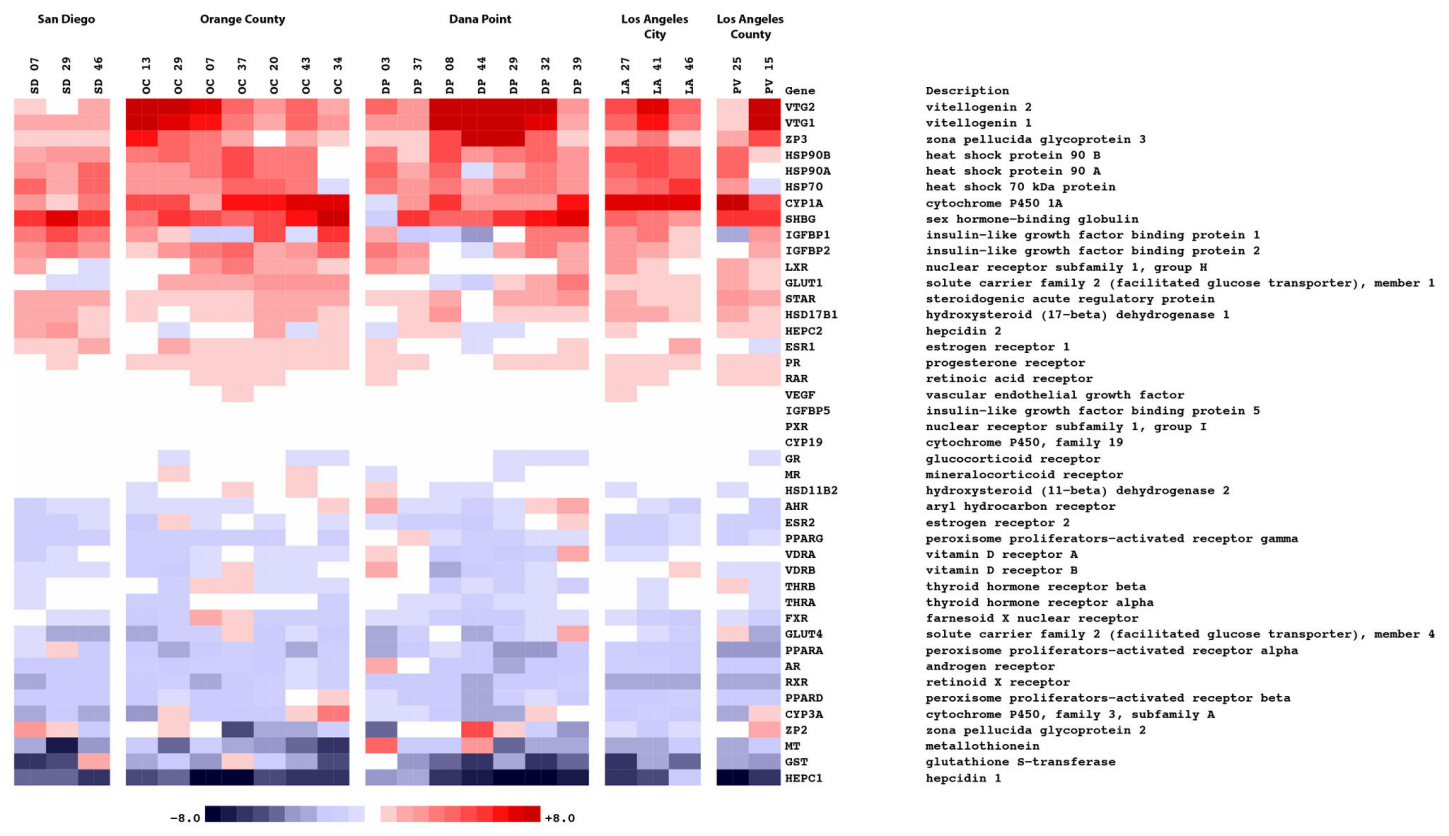
Multi-species microarray gene expression profiling of turbots for exposure to endocrine disruptors. The mRNA level fold changes observed between exposed and control fish are depicted as a diagnostic heat map representative of all the genes present on the multi-species array. The log_2_ ratio for each gene is defined as the mean of all representations of that gene on the microarray; these include probes from different regions of the gene, as well as from different species of fish (individual log_2_ ratios for each unique probe are calculated as the median of 46 replicates present on the array). The range of colors is between -8-fold and +8-fold and preserves qualitative relationships among individual values. All fold changes outside of this range have been truncated to ± 8.

Gene expression changes in all the genes represented on the multi-species array (Table S1) were also carefully investigated in the field fish from the four treated municipal wastewater discharges and one field reference site. The mRNA level fold changes observed between these exposed fish and the laboratory reference fish are depicted as a diagnostic heat map (Figure 3). We plotted the data in this manner so that whenever one group’s overall expression was higher than the other’s, for example exposed fish, the former was colored in prevalently red tones (increased expression) whereas the latter was colored in blue tones (decreased expression). The white values in the middle of the heat map correspond to transcripts whose expression levels remained unchanged.

Fish sampled at all field sites exhibited strong decreases in the expression of many genes relative to the reference fish. Transcripts encoding peroxisome proliferator-activated receptors (*PPARα* and *PPARγ*) were down-regulated relative to laboratory reference fish. Additionally, thyroid receptor α (TRα), androgen receptor (AR), retinoid X receptor (RXR), Hepcidin (*HEPC*), Cytochrome P450, family 3, subfamily A, (*CYP3A*), glutathione S-transferase (GST), metallothionein (MT), estrogen receptor 2 (*ERβ*/*ESR2*), and the H2 gene mRNA were also down-regulated in the majority of fish from all the sites. Vitellogenin 1 and 2 (*Vtg1, Vtg2*) were up-regulated in fish from all sites studied, except in one fish sampled from the San Diego site which had no change in *Vtg2* expression. Cytochrome P450, family 1, subfamily A (*CYP1A*) mRNA was up-regulated in all male fish except in one fish sampled from Dana Point (DP 03). Zona pellucida glycoprotein 3 (*ZP3*), sex hormone-binding globulin (SHBG), heat shock protein 90 (*HSP90A, HSP90B*), heat shock protein 70 (*HSP70*) all revealed altered mRNA expression levels in the majority of fish across the different sampling sites.

### Quantitative RT-PCR analysis of turbot gene expression

In order to use Q-PCR to determine if the microarray data was accurately monitoring changes in hepatic gene expression in the hornyhead turbot, we cloned, via reverse transcriptase PCR, partial fragments corresponding to highly conserved regions in 5 target genes of interest, namely *Vtg1, Vtg2, CYP1A, ERα/ESR1* and *ERβ/ESR2*. Identities of the fragments were confirmed by Sanger DNA sequencing. The primer sequences used for PCR are provided in the Supporting Information (Table S2). The sequence data have been deposited into GenBank (accession numbers FJ042791-FJ042800). These short hornyhead turbot-specific sequences obtained using multi-species conserved primers were used for SYBR green quantitative PCR experiments on ten individual turbot sampled from the field. After real time PCR amplification, a melt curve was carried out, in which the temperature was raised by a fraction of a degree and the change in fluorescence was measured. This revealed similar peaks in all the samples indicating that a specific DNA fragment corresponding to the predicted size was detected.

**Figure 4:**
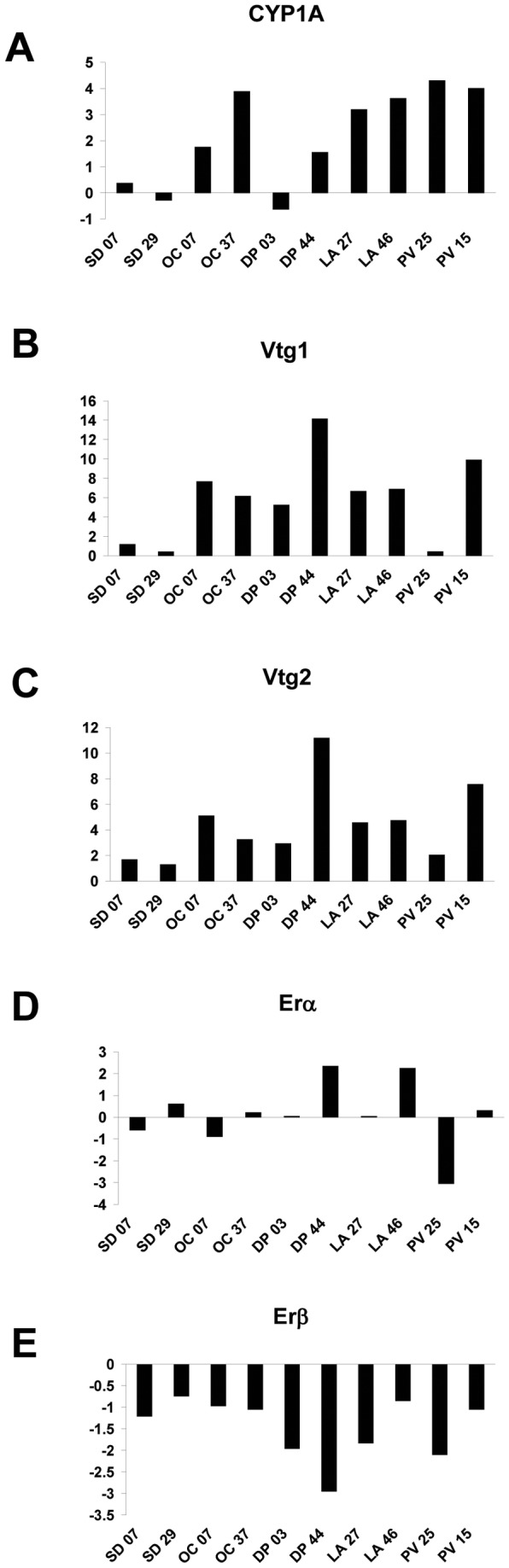
Q-PCR validation of Multi-Species Endocrine Microarray. Multi-species SYBR green Q-PCR validation of multi-species microarray for (A) *CYP3A* (B) *Vit1* (C) *Vit2* (D) ESR1/*ERα* (E) ESR2/*ERβ* specific transcripts. *GAPDH*-normalized fold changes (based on triplicate measurements) of gene expression in turbot liver from selected impacted sites with respect to reference fish are presented. Each fold change was derived from the mean log_2_ ratio between each fish and a reference derived from two control fish. *Vit1* and *Vit2* transcripts were strongly up-regulated in all fish. *ERα* was down-regulated in one fish and up-regulated in two others relative to control fish. *ERβ* was down-regulated in all fish examined relative to control fish. *CYP3A* was up-regulated in eight and down-regulated in two fish.

The Q-PCR based gene expression profiling of hornyhead turbots from selected field sites is presented in Figure 4. All data are presented as GAPDH-normalized fold changes of gene expression in the hornyhead turbot liver from impacted sites with respect to reference fish. The fold change data shown were derived from the mean log_2_ ratio between each fish and two laboratory reference fish. The fish used as a reference for these Q-PCR studies were the fish used for microarray experimentation, designated A and C as described above and previously housed in a clean-water laboratory setting for four weeks. The dynamic range of the fold changes observed with the Q-PCR analysis was as anticipated much greater than with the microarray analysis. However qualitative agreement was generally observed with the direction of the fold changes. *ERβ/ESR2* was strongly down-regulated in all samples, *Vtg1* and *Vtg2* transcripts were up-regulated in all samples, with greater than a 6-fold induction observed in six hornyhead turbots. *CYP1A* was up-regulated in seven fish. Thus, these Q-PCR assays validate the microarray analysis for these genes in these fish.

### Phenotypic Correlations

Significant Spearman correlations were found between the fish phenotypic characteristics (e.g., weight) and the expression of 24 genes. These correlations showed relationships between phenotypic changes in the fish (e.g., changes in the plasma concentration of hormones) and the genotypic responses observed. Spearman correlation r values ranged from -0.6 to 0.7, while p values ranged from < 0.0001 to 0.05 (Table 3). Genes related to endocrine functions, xenobiotic metabolism, development, growth, as well as immune and stress responses correlated with fish size. The gene expression of the *RXR*, *HEPC2*, *IGFBP1*, *IGFBP2*, *SHBG*, and *STAR* genes was lower in fish that had a larger length. While the expression of the *HSP90B*, *MT*, *Vtg1*, *Vtg2*, and *ZP3* genes had a positive correlation with fish length (Table 3). The expression of the *IGFBP1*, *RXR*, *SHBG*, *IGFBP2*, and *STAR* genes was lower in organisms with higher weight. There was a positive correlation among the expression of the *CYP1A*, *MT*, *Vtg1*, *Vtg2*, *HSP90B* and *ZP3* genes and the weight of the fish. Correlations of the thyroid receptors (*TRα/β*) with *ZP2* and *ZP3*, *Vtg1* and *Vtg2*, nuclear receptors (*FXR* and *LXR*) and *HSP70* were found. The correlations were negative for *ZP2, ZP3, Vtg1* and *Vtg2* and positive for the nuclear receptors and stress response transcripts (Table 3).

**Table 3:** Significant Spearman correlation analysis between gene expression data for *Vtg1, Vtg2, ZP3, TRα*, *TRβ* and defined phenotypic endpoints or other differentially regulated transcripts.

**Gene**	**Variable**	**Spearman coefficient**	***Pvalue***
*Vtg1*	Calculated Age (y)	0.5	0.026
*Vtg1*	SL (cm)	0.5	0.026
*Vtg1*	Weight (g)	0.5	0.014
*Vtg1*	*Vtg2*	0.5	< 0.0001
*Vtg2*	Calculated Age (y)	0.5	0.012
*Vtg2*	SL (cm)	0.5	0.013
*Vtg2*	Weight (g)	0.5	0.011
*ZP2*	Cortisol (ng/ml)	-0.4	0.043
*ZP2*	*GST*	-0.5	0.013
*ZP2*	*ZP3*	0.5	0.036
*ZP3*	Cortisol (ng/ml)	-0.5	0.02
*ZP3*	Calculated Age (y)	0.6	0.005
*ZP3*	SL (cm)	0.6	0.004
*ZP3*	Weight (g)	0.6	0.003
*ZP3*	*Vtg1*	0.9	< 0.0001
*ZP3*	*Vtg2*	0.9	< 0.0001
*TRα*	FXR	0.7	0.0005
*TRα*	*HSP70*	0.4	0.0394
*TRα*	*HSP90A*	0.6	0.0042
*TRα*	*LXR*	0.7	0.0005
*TRα*	*TRβ*	0.7	0.0005
*TRα*	*VDRα*	0.5	0.015
*TRα*	*VDRβ*	0.7	0.001
*TRα*	*Vtg1*	-0.5	0.0236
*TRα*	*Vtg2*	-0.4	0.0512
*TRα*	*ZP2*	-0.6	0.0056
*TRα*	*ZP3*	-0.5	0.017
*TRβ*	*FXR*	0.6	0.0041
*TRβ*	*HSP90A*	0.5	0.0211
*TRβ*	*LXR*	0.5	0.0247
*TRβ*	*SHBG*	-0.5	0.0331
*TRβ*	*VDRα*	0.5	0.0243
*TRβ*	*VDRβ*	0.6	0.0028
*TRβ*	*Vtg2*	-0.4	0.0585

Additional significant Spearman correlations were found with hormones and the gene expression and this data is provided in the supporting information (Table S3). Specifically there was a negative correlation between E2 levels and expression of *GLUT1*, *MR* and *PR* and a positive correlation with *PPARγ* expression. A negative correlation was found between IGF-1 levels and expression of *AHR*. A positive correlation was found between IGF-1 levels and expression of *ZP2*. A positive correlation was found between testosterone levels and expression of *PPARα*. A positive correlation was found between VTG protein levels and expression of *GR*, *HSD17β1*, *MR*, *PPARα*. There was a positive correlation between cortisol levels and expression of *ESR1*, *HSD11β2*, *HSP70* and *HSP90A*. A negative correlation was uncovered between cortisol levels and expression of *ZP2* and *ZP3*.

### Sediment Analysis

Samples of sediment from the sites where the hornyhead turbot were collected have been analyzed previously for a number of legacy and emerging chemicals [21,22]. A suite of 89 legacy and emerging contaminants were measured in the sediments. Several contaminants were found at detectable levels amongst them legacy organochlorine pesticides and personal care compounds. Many of the detected compounds such as DDTs or triclosan are known to elicit endocrine and stress responses [21]. As seen in Table 2, several of the sediments contained 4-nonylphenol, an estrogenic chemical, polybrominated diphenyl ethers (PBDEs), which are flame retardants, and polychlorinated biphenyls (PCBs). The sediment obtained from Dana Point indicated that it was the least contaminated site of those examined. Based on the physiochemical properties of these analytes, chemicals present in these sediments would be expected to enter the food chain of the turbot.

## Discussion

In recent years, as more knowledge has accumulated about the effects of chemicals such as BPA, organotins and phthalates, it has become clear that endocrine disruption is complex and involves the altered expression of many genes [2,4,7]. An important advance in detecting the effects of xenobiotics was the development of high-density DNA microarrays or biochips [23-25]. This technology provides a sensitive and comprehensive snapshot of alterations in endocrine responses in fish that may be exposed to low levels of endocrine disrupting chemicals [19,26-28]. Microarrays enable analysis of complex environmental chemical mixtures by providing transcriptomic profiles or signatures based on the contaminants present. However, it is important to link the molecular responses with physiological changes to determine if the responses at the gene expression level can cause down stream biological effects. We used an optimized microarray tool to examine gene expression in hornyhead turbot, a species which has not yet had its genome sequenced and annotated. This microarray is an improvement over an earlier microarray tool, which was developed using multi-species probes and validated by studying gene expression profiles in zebrafish, a species that is phylogenetically distant from turbot. This earlier work revealed that our multi-species microarray approach was suitable for measuring endocrine responses in turbot and other fish [19]. The rationale for use of 60-mer oligonucleotides in the second generation platform described here is that they provide more specificity than cDNA-based microarrays accommodate sequence differences and species specific codon usage [29-31]. The novelty of this platform is that it used highly conserved probes from several fish species, permitting application of the array to studies involving hornyhead turbot and zebrafish [19].

We compared data output from similar samples assayed with the prototype array reported previously [19] and the second generation microarray. This revealed that genes that are up-regulated have a greater chance of being detected with the prototype array than those that are down-regulated. The second generation microarray is a major improvement in that it does not have this limitation (Figure 1E).

After these initial optimization and platform comparison experiments, we assessed alterations in hepatic gene expression in male hornyhead turbots collected during a collaborative marine monitoring study in 2006 at five field stations in southern California that vary in magnitude of contaminant exposure. Our objective was to use the microarray tool to assess gene expression levels for key biomarkers of endocrine disruption. Levels in plasma of vitellogenin, testosterone, estradiol, cortisol and thyroxine were determined in the fish selected for microarray analysis [Table 1] [19,32-34]. Table 1 reveals that there is substantial variation in hormone levels among the fish collected from different sites and between fish from the same site. This may be due to a combination of factors including genetic heterogeneity in wild fish, variation in the age and life histories of the fish as well as variations in exposure to chemical contaminants, diet and reproductive status [1,35,36]. This variability among the fish in levels of plasma hormone and vitellogenin levels posed a challenge for evaluating the exposure of fish from the five sites to endocrine disruptors.

To get another metric for the presence of pollutants at the five sites in southern California, we used the multi-species microarray to investigate hepatic gene expression in these fish. The liver was our primary focus because it is the key organ involved in detoxification. The improved multi-species array contained expanded probe content (Table S1) including targets with defined roles in endocrine pathways and processes [6,37-40], in addition to well-defined biomarkers for contaminant exposure [41]. Microarray analysis detected differences in hepatic gene expression patterns in male hornyhead turbot from all five areas compared to laboratory reference fish. We focused on the salient changes in expression patterns below.

The majority of the field hornyhead turbot examined showed up-regulation of *Vtg1* and *Vtg2, ZP3* and *SHBG*. *SHBG* binds 17β-estradiol and ethynylestradiol, a synthetic estrogen that is a contaminant of emerging concern [42,43]. *SHBG* was up-regulated in all but one fish. Up-regulation of vitellogenin and *ZP3* transcripts in male fish is a well-established response to estrogens, indicating that these fish may have been exposed to environmental xenoestrogens, anti-androgens or a contaminant that up-regulates steroidogenesis thereby increasing endogenous estrogen levels [6,44]. This interpretation is supported by chemical analysis of sediments from the sites where the fish were captured, which indicate the presence of nonylphenol, a xenoestrogen [21,22]. Interestingly males which experienced the greatest fold change in *Vtg1* and *Vtg2* also experienced the greatest fold change in *ZP3*. Fish with little or no fold change in *Vtg* expression also experienced little or no fold change in *ZP3* expression. The data suggest that *Vtg1, Vtg2* and *ZP3* co-vary with one another and that *Vtg1* and *Vtg2* are more responsive than *ZP3* as previously observed with *Gobius* *Niger* [45]

The levels of plasma VTG protein and expression of *Vtg* mRNA in fish collected from the five field sites do not always agree. This was not unexpected because plasma VTG could be due to exposure to a xenoestrogen or anti-androgen several days prior to collections, while *Vtg* expression is a measure of a response to near-term exposure to chemicals. Moreover, high levels of plasma VTG may have a negative feed-back on *Vtg* transcription. Nevertheless, the global picture of vitellogenin synthesis from plasma measurements, microarray analysis and Q-PCR indicates that most of the field-caught fish were exposed to either xenoestrogens or anti-androgens or both. Importantly, microarray analysis of *Vtg* and *Zp3* expression and Q-PCR analysis of *Vtg* gene expression clearly finds a lower magnitude of estrogenic response in a male from Palos Verdes [PV25] and two males from San Diego (Figure 4), demonstrating the utility of molecular methods for evaluating endocrine responses and exposures in the environment.

There was a robust correlation between expression of *Vtg* and *ZP* mRNAs (r=0.99; p <0.001). This is reasonable because both genes are biomarkers for xenoestrogens and 4-nonylphenol; a plastic degradate detected in the sediments from where the turbot were collected. Correlation analysis also revealed that expression of both genes showed very strong significant association with age, weight and length of the fish. Furthermore, we found negative correlations of thyroid receptors and VTG/ ZP transcripts (r ranged from -0.5 to -0.4; p< 0.05), which could in part explained the lower thyroxine concentrations found in the plasma of these fish [46].

There are differences in expression of *ERα* and *ERβ* among the different fish. The trend was decreased expression of *ESR2*/*ERβ* in the majority of fish, while two fish, OC29 and DP39 showed increased expression and expression remained unchanged in OC37, 0C43 and DP32. For *ESR1*/*ERα* the trend was increased expression in many of the fish with decreased expression in DP44. DP37, DP08, DP29, DP32, LA27, LA41, PV25 had similar expression levels of *ESR1*/*ERα* to control fish. The functions of ERα and ERβ in fish are not fully understood. In mammals, ERα and ERβ appear to have some opposing physiological activities in many organs [47,48]. That is, activation of ERα promotes cell growth, while activation of ERβ inhibits cell growth. Despite their sequence similarity, there are differences in the binding and transcriptional response of human ERα and ERα for some chemicals [49,50]. ERβ expression was dominant at the time of collection from the field sites.

*CYP1A* mRNA was up-regulated in all but one of the fish examined. The heat shock proteins *HSP90A*, *HSP90B* and *HSP70* all revealed elevated mRNA expression levels in the majority of fish from the different sampling sites. These genes are biomarkers for exposure to compounds such as planar organic hydrocarbons or to stress. Many of the compounds detected in the sediment such as DDTs or triclosan are known to elicit endocrine and stress responses and may contribute to the altered expression of heat shock proteins [21]. Interestingly, the OC34 and PV 15 fish from SD had low expression of *CYP1A*, *HSP70* and *HSP90*.

The majority of the exposed turbot also exhibited down-regulation of *CYP3A*. We previously noted a modest repression of *CYP3A* in zebrafish exposed to 4-nonylphenol and a strong repression following estradiol exposure [19]. Similar results have been reported in trout [41,51], suggesting an important role of sex hormones in *CYP3A* expression.

Other mRNA targets impacted in exposed fish included the peptide hormone Hepcidin (*HEPC1/HAMP1*), Metallothionein (MT) and Glutathione S-transferase alpha (*GSTα*). Hepcidin is a peptide hormone produced by the liver [52,53]. Hepcidin is a negative regulator of iron absorption and mobilization. Thus, low levels of hepcidin promote iron absorption, and are indicative of an iron deficiency. Hepcidin also serves as an antimicrobial peptide [54]. *HEPC1* was down-regulated strongly in all fish examined relative to the reference fish. This could be due to lower oxygen in the water in the field sites compared to the oxygen in the clean-water laboratory setting. Another potential cause for lower hepcidin levels could be exposure to a xenoestrogen because Robertson et al. [55], recently demonstrated that exposure to estradiol decreased expression of hepcidin-1 and blocked the induction of hepcidin-2 expression by bacterial exposure in largemouth bass. This suggests that exposure of hornyhead turbot to either xenoestrogens or low oxygen levels may make fish more susceptible to disease by blocking production of hepcidin.

Metallothioneins (MT) have an important role in detoxification of essential metals [e.g. Cu, Zn] and non-essential metals [e.g. Ag, Cd, Hg] in fish, in which *MT* synthesis is induced by metal contaminants in the environment [56,57]. This contrasts with the results of the multi-species microarray analysis of *MT* mRNA in the field hornyhead turbot, in which *MT* expression was strongly down regulated. A possible explanation for our data may be found in studies showing that bisphenol A and nonylphenol suppressed *MT* gene expression in the mangrove killifish ( *Kryptolebias* *marmoratus* ) [58]. Inhibition of MT expression by ethinylestradiol in the liver of lake trout ( *Salvelinus* *namaycush* ) also has been reported [59]. These data with killifish and lake trout are consistent with exposure of the field hornyhead turbots to either xenoestrogens or anti-androgens.

Glutathione S-transferases (GSTs) are a family of xenobiotic metabolizing enzymes [40]. GSTs detoxify endogenous and exogenous substances (drugs, pesticides, and other pollutants) through their conjugation to glutathione (GSH). We find that *GSTα* mRNA was strongly down regulated in almost all of the fish studied. Down-regulation of *GSTα* was surprising because pollutant exposure should lead to increased levels of *GST*. However, there is evidence for down-regulation of *GST* under some conditions. For example, GST*α* is down-regulated in goldfish exposed to microcystin, a cyclic polypeptide that is a heptotoxin [60]. Also EE2 down-regulated GSTπ in Atlantic salmon [44].

## Conclusions

Together, the results obtained using the multi-species microarray and Q-PCR to assess the endocrine status of male hornyhead turbots in five coastal field stations indicate that these fish were exposed to a mixture of endocrine disruptors capable of interacting with the estrogen and thyroid responses. Despite the genetic heterogeneity of these wild fish and differential exposure to chemicals, food and environment the multi-species microarray identified differences in gene expression among fish captured from different field sites. These results validate this tool for comparisons of endocrine-disrupting contaminants at different sites and highlight the utility of the multi-species microarray approach as a sensitive diagnostic for the presence of endocrine disruptors in the aquatic environment.

## Materials and Methods

### Ethics Statement

The fish specimens used in this study were hornyhead turbot ( *Pleuronicthys* *verticalis* ), a species frequently used in California monitoring programs. All tissue samples were obtained from fish collected by trawl from California coastal waters. Fish collections were approved by and conducted under scientific collection permits issued by the California Department of Fish and Wildlife. This species is not threatened or endangered, and the collection sites were not located in ecological reserves or areas receiving special ecological protection. The sampling method (otter trawl) limits bycatch mortality by using a relatively small net, holding the catch in flowing seawater, and promptly returning non-target individuals to the ocean. These methods are the same as those used in regional monitoring programs, which have been approved by local and federal fish and wildlife and regulatory agencies.

Humane handling of the fish was assured by the use of a Standard Operating Procedure developed specifically for the study and approved by a Steering Committee composed of the study participants. This Steering Committee included research faculty from universities having established IACUCs and senior management from public utility departments. SCCWRP does not have an IACUC committee with authority to approve animal care procedures, because such a committee is not required by its activities or funding sources and fish are not USDA-covered species.

All fish used were treated humanely and with regard for alleviation of suffering. Multiple precautions were followed to minimize stress and suffering of the specimens, including:

- Trawls were of short duration (5-10 minutes) and at low speed to limit stress during capture. Studies have shown that fish move freely within the net during trawling.
- Fish were transferred rapidly from the net to holding tanks with flowing seawater. The water was oxygenated and at a similar temperature to that of the fish’s habitat.
- Fish were anesthetized with MS222 prior to handling. Fish were in a state of deep anesthesia (unresponsive to touch) prior to removal from the water and collection of blood.
- Fish were sacrificed by cervical dislocation prior to dissection. This procedure was conducted while the fish were still under anesthesia [22]. The livers were harvested and frozen in liquid nitrogen and stored at -70°C.

### Steroid hormone, thyroxine and vitellogenin assays

Plasma concentrations of steroid hormones and thyroxine levels and vitellogenin protein levels were measured as described in [19,34,61]. The methods are outlined below.

### Hornyhead Turbot vitellogenin assay

Wells were coated with 100µl of 0.8µg/ml California Halibut VTG (provided by Amanda Palumbo of UC, Davis) in 50mM carbonate buffer. Non-specific binding wells were coated with 1% non-fat milk in 50 mM carbonate buffer. Plates were then incubated at 37C for 2h. Wells were washed three times with 10mM Tris-phosphate buffer saline (TPBS), then blocked with 200µl of 2% non-fat milk in TPBS for 45min at 37C. The wells were then washed again three times with TPBS. Standards (purified Halibut VTG) and samples were diluted in TPBS. Primary antibody (rabbit anti-Turbot VTG purchased from Cayman Chemical, Ann Arbor, MI) diluted in TPBS was added to standards and samples at a ratio of 1:1, for a final concentration of antibody of 1:1000. These solutions were then incubated for 2h at 37C. One hundred microliters of each solution was then added in triplicate to the wells and incubated again for 2h at 37C. The wells were then washed three times with TPBS. The secondary antibody (goat anti-rabbit labeled with alkaline phosphatase purchased from Biorad in Hercules, CA) was diluted to 1:2000 in TPBS then added to the wells and incubated for 45 min at 37C. The wells were washed twice with TPBS and once with PBS. The substrate *p*-nitrophenylphosphate diluted in diethanolamine buffer was added to each well at volume of 100µl. The plate was then incubated for about 1h in dark. The absorbance was measured with a microplate reader at a wavelength of 405nm.

### Measurement of plasma concentrations of steroid hormones

Plasma concentrations of 17Î²-estradiol, testosterone, and cortisol were measured by specific radioimmunoassays using ^125^I-labeled steroid and polyclonal rabbit antisera obtained from DSL/Beckman Coulter (Webster, TX). Separation of free and bound antigen was achieved using a double antibody system (goat anti-rabbit gamma globulin serum) and polyethylene glycol as a precipitating aid. Counts per minute (cpm) of antibody-bound ^125^I-steroid were measured in a PerkinElmer Cobra II gamma counter (Packard Instruments Co., Boston, MA). The standard curves were utilized to calculate concentrations of hormone in the unknowns using SigmaPlot 8.0 software (Four-Parameter Logistic Curve Function, SPSS Inc., Chicago, IL). Estimated coefficients of variation are between 6.1-7.5 (intra-assay) and 8.0-9.4 (inter-assay) for both assays

### Hornyhead turbot sample collection

A new cohort of male hornyhead turbot was collected for this project as part of a southern California collaborative marine monitoring study in May and June 2006. Table 1 shows steroids and thyroxine levels in fish collected from five field sites near outfall discharges and a reference area. Chemical analysis for selected compounds detected in sediments from these areas is presented in Table 2.

Fifty fish were collected from each of five sites described in the text, which included sites near the four largest ocean municipal wastewater discharges in southern California (240 to 440 x 10^9^ L/day) and a reference site distant from discharges. Owing to cost considerations, fifty microarrays per site were not possible. Livers from ten male fish from each site were selected for measurement of gene expression and the RNA assessed for quality using Agilent Bioanalyzer analysis. Only RNA with a RIN (RNA integrity number) >6 were considered for microarray analyses. In the case of LA County only two fish yielded RNA that met this strict criteria, i.e. a RIN value >6. In addition, we used livers from three reference fish, obtained from a separate monitoring station near Dana Point, CA, from a relatively non-impacted area and maintained in a clean-water laboratory setting for four weeks. Dana Point (DP) was chosen because it is reasonably far from the main municipal wastewater discharges investigated in the present study, and because previous studies have used it as a reference site [22,62]. Collected animals were sacrificed immediately following capture and the livers were harvested, frozen in liquid nitrogen or dry ice and stored at -70 °C.

### Design of the second generation multi-species array

A challenge in developing this platform was the scarcity of hornyhead turbot sequence data in GenBank. Although DNA sequences have been described for the European turbot *Scophthalmus* *maximus* [17,18] our objective was to develop a tool for a molecular analysis of endocrine disruption in a variety of fish species living in marine or freshwater environments that are exposed to wastewater effluent and pesticides. Thus, we collected conserved sequences for genes from many species rather than use probe sequences specific to one organism.

To construct the multi-species microarray, we queried GenBank for sequences in a variety of fish to uncover regions of sequence conservation that would permit design of a microarray suitable for detecting altered gene expression in multiple fish species. This approach was feasible as the complete genome sequences for two Tetraodontiformes, *Fugu* and *Tetraodon* , were available. Moreover, many genes from various Perciformes were present in GenBank. Tetraodontiformes and Perciformes are phylogenetically close to Pleuronectiformes as demonstrated in Figure S1. The complete genome sequence of zebrafish, a distant relative of Pleuronectiformes also helped guide our efforts. We hypothesized that sequences highly conserved in *Danio, Fugu,* *Tetraodon* and various Perciformes were likely to be conserved in hornyhead turbot.

We designed *in silico* a 60-mer oligonucleotide-based multi-species microarray using the eArray – Custom Microarray Design Application from Agilent (Santa Clara, CA). The oligonucleotide probes were based on conserved sequences from genes of interest. For example in the original microarray oligonucleotide probes were designed by collecting available fish sequences in GenBank for a given gene (e.g. *ESR1/ERα*, *Vtg*, *CYP3A* and *FXR*) using BLAST [63].

The rationale for use of 60-mer oligonucleotides in the second generation platform described here is that they provide more specificity than cDNA-based microarrays accommodate sequence differences and species specific codon usage [29-31]. The novelty of this platform is that it used highly conserved probes from several fish species, permitting application of the array to studies involving hornyhead turbot and zebrafish [19].

The “second generation” microarray included genes involved in metal binding and detoxification (metallothionein [MT], the aryl hydrocarbon receptor [AhR]), genes involved in growth and development (vascular endothelial growth factor [VEGF]), reproduction (sex hormone-binding globulin [SHBG], zona pellucida protein 2 [*ZP2*] and zona pellucida protein 3 [*ZP3*], stress responses (heat shock proteins [*HSP70, HSP90*]), oxidative stress responses (glutathione S-transferase [GSH]) and iron metabolism (hepcidin [HEPC]).

Sequences from *Tetraodoniformes* (Fugu, Tetraodon) and *Perciformes* (cichlid, tilapia, sea bass, seabream), which are close from a phylogenetic perspective to *Pleuronectiformes* (hornyhead turbot, California halibut) were selected (see Figure S1). Available sequences from other species including medaka, stickleback and zebrafish also were used, in addition to hornyhead turbot-specific cDNA sequences obtained via degenerate PCR. Multiple alignments were constructed using Clustal X to uncover conserved regions [64] and nucleotide sequences were analyzed using Oligowiz [29,59] to design 60-mer microarray probes. Each copy of an individual gene from several fish species was subjected to a pair-wise BLAST comparison with the corresponding gene from other fish to insure that the DNA sequence contained 80% and 90% identity thereby increasing the likelihood that the homologous turbot sequence would contain at least 85% identity to one of the oligonucleotides. The gene names and corresponding gene symbols are provided in the Supporting Information (Table S1).

In total 326 unique probes were present on the array, each of which was replicated in 46 distinct spatial locations throughout the array. The array design (Aquatic Multi-species Array v2.0) has been deposited in the ArrayExpress Database (accession numbers A-MEXP-2291) (European Bioinformatics Institute 2013).

### RNA extraction, fluorescent target labeling and microarray hybridizations

RNA extraction labeling and hybridization to the multi-species microarray were carried out in accordance with single color Agilent hybridization protocols and have been described previously [19]. Essentially isolation of total RNA from liver samples was performed using TRIzol reagent (Invitrogen) and the extracted RNA were further purified using the RNeasy Mini kit (Qiagen, Valencia, CA). All RNA was and treated with DNase. The concentrations were determined by absorbance readings (OD) at 260nm using an ND-1000 (Nanodrop, Wilmington, DE). RNA was further assessed for integrity with the 6000 Nano LabChip assay from Agilent, (Palo Alto, CA). 100 ng of total RNA were converted into fluorescently labeled Cy 3 cRNA using the Low RNA Input Fluorescent Linear Amplification Kit (Agilent). Fluorescent targets were purified to remove unincorporated nucleotides using RNeasy (Qiagen). Absorbance (OD) at 260nm was used to quantify the cRNA concentrations, and absorbance at 550nm was used to measure the efficiency of Cy3 dye incorporation. An incorporation efficiency of 9 pmol/µg or greater was deemed necessary before proceeding with hybridization. 1 µg of fragmented cRNA for each sample, were hybridized to the array in accordance with single color Agilent hybridization protocols. The hybridization conditions were such that the hybridization was carried out under high salt conditions to facilitate specific probe and target interactions. Data were collected using an Agilent Microarray Scanner and Feature Extraction Software (v10.5). Array data has been deposited in the ArrayExpress Database (accession number E-MEXP-3852) (European Bioinformatics Institute 2013).

### Microarray data analysis, statistical testing, sample size and related variables

The power of a statistical test is the probability that the test (correctly) decides that there is a difference in gene expression when there truly is a difference. The quantities that determine power is the sample size or number of replicates, the effect size, and the alpha level, which are the probability of detecting the effect when in fact there isn’t one (i.e., type I error rate). We utilized the pwr.norm.test function of the pwr library for R (R Development Core Team, 2005) [65,66] to determine the number of replicates needed. We determined that for an outbred population a biological replicate value of 3 has sufficient power to detect fold changes of 1.5 and 2 with power values of 0.7 and 0.99 respectively. Where available we used minimally three and if possible additional impacted fish per site; San Diego (three fish), Orange, County (seven fish), Dana Point (seven fish), Los Angeles City (three fish). Only two fish were available from Los Angeles County.

Statistical analysis of the microarray experiment involved two steps: normalization of microarray data, and sorting of the genes according to interest. We normalized all samples simultaneously using a multiple-loess technique described previously. For array based analyses of this nature this global normalization technique is more appropriate than utilization of individual housekeeping probes that are not expected to change from one condition to another [19,20]. To investigate alterations in gene expression of controls and exposed fish, we used two independent analytical methods, MA plots and box plots. MA plots facilitated visualization of the intensity-dependent ratio of the microarray data, where M is the intensity ratio and A is the average intensity for a given data point in the plot. Box plot or box-and-whisker diagrams were used to graphically depict the microarray data through five-number summaries: the smallest observation (sample minimum), lower quartile (Q1), median (Q2), upper quartile (Q3), and largest observation (sample maximum). Heat maps were constructed using an in-house software program that implements Ward clustering. The clustering was not utilized to find any particular pattern in the data. Its primary purpose was to visually consolidate groups of genes as found by the means described above.

### Amplification, sequencing and Q-PCR of hornyhead turbot mRNAs

We amplified partial turbot transcripts using conserved sequences from other fish species to guide the choice of primer design. Gene-specific primers were designed using OligoWiz software [29]. All the amplicons were directly sequenced using the respective forward and reverse PCR primers. Sequencing reads were subjected to a series of quality control measures, including a phred quality score >20, and manual trace inspection. The identity of each sequence was confirmed by BLAST searches. These sequences have been deposited into GenBank (accession numbers FJ042791-FJ042800). Primer sequences are outlined in the Supporting Information (Table S2).

### Quantitative real-time PCR analysis

Relative turbot liver mRNA transcript levels were measured by real-time quantitative RT-PCR in a LightCycler 480 as described previously [19]. RNA was DNAse treated and negative RT controls without reverse transcriptase addition were included. Amplification and melting curves were carefully examined for all assays. We investigated *GAPDH* and *β-actin* as reference transcript for these particular studies and noted that both performed equally well in Q-PCR experiments. Both were detected with a Cp value of approximately 26. We opted to use *GAPDH* to normalize all expression values, although *β-actin* would have been equally suitable. Each sample was run in triplicate and mean values were reported. Normalized gene expression values were obtained using LightCycler Relative Quantification software. Relative gene copy numbers were derived using the formula 2ΔCT where ΔCT is the difference in amplification cycles required to detect amplification product from equal starting concentrations of turbot liver RNA.

### Spearman correlation analysis

Differential gene expression and phenotypic characteristics such as plasma protein levels and fish size were correlated to investigate relationships (Table 3). Spearman correlations were calculated using JMP version 8 (SAS Institute, USA). Data of all the males from the five studied stations were used in this analysis.

## Supporting Information

Figure S1 Flatfish (*Pleuronectiformes*) in an evolutionary context. Adapted from the phylogeny at http://cichlidresearch.com/fish_html/cactinop.html (Nelson 2006). Tetraodontiformes (Fugu, Tetraodon) and Perciformes (cichlid, tilapia, sea bass, seabream, perch) are close phylogenetic relatives of Pleuronectiformes (turbot, halibut, sole).
(PDF)

Figure S2 The probes were concentrated along a line, evidence that this represents a microarray artifact and that caution is warranted when performing differential expression measurements using microarrays. (PDF)

Figure S3 Examination of the feature intensity distribution in control and impacted fish livers. The X-axis represents microarray data (M) corresponding to three pooled control fish samples (A,B,C), the three individual control samples A, B, and C respectively and exposed fish sampled from sanitation districts in San Diego, Orange County, Dana Point, Los Angeles City and Los Angeles County. M specifically is a measure of differential gene expression (log_2_ (exposed /pooled control ABC) for all these samples. Only genes considered detected were depicted in these plots. The fish from impacted areas exhibited wider distributions in differentially expressed genes and outliers indicating that a large number of genes are differentially regulated with regard to control fish. Boxes represent the inter-quartile range, with the 75th percentile at the top and the 25th percentile at the bottom. The line in the middle of the box represents the 50th percentile, or median. Whiskers represent the rest of the distribution, with their terminations at 1.5 times the inter-quartile range in either direction. The individual notches represent outliers, i.e., unusually strongly modulated genes.
(PDF)

Figure S4 Feature level analysis of the top 100 differentially regulated multi-species probes. The fold changes were determined from log_2_ ratios between the probe signal of each fish and that of the pooled control sample. There were 326 unique sequence probes on the array, each of which was replicated in 46 distinct locations. The log_2_ ratio was calculated for each probe as the median of the 46 replicate log_2_ ratios. The 326 unique probes were subsequently sorted by their importance in descending order of the sum-squared statistic (i.e., sum of squares of log_2_ ratios across all fish). The top 100 probes were selected and included in this heat map. Since the majority of genes were represented on the microarray by sequences derived from several fish species, we further consolidated probes from the same gene, albeit different species, into a color strip of the same width. The range of colors is between -8-fold and +8-fold and preserves qualitative relationships among the individual values. All fold changes outside of this range have been truncated to ± 8.
(PDF)

Table S1 There are 37 diagnostic (endocrine) markers on the array. The gene and corresponding gene symbols are outlined.
(PDF)

Table S2 SYBR green qPCR validation was carried out for *CYP1A, Vtg1, Vtg2, ERα* and *ERβ* specific transcripts. The oligonucleotide probe sequences and the corresponding amplicon sizes are given.
(PDF)

Table S3 Expanded significant Spearman correlation analysis between gene expression data and defined phenotypic endpoints or other differentially regulated transcripts. (XLS)
