# Supplementary material for: Molecular Analysis of Endocrine Disruption in Hornyhead Turbot at Wastewater Outfalls in Southern California Using a Second Generation Multi-Species Microarray
Source: PLoS One. 2013 Sep 25;8(9):e75553. doi: 10.1371/journal.pone.0075553 (PMC3783431; doi:10.1371/journal.pone.0075553)
Supplement: Figure S1 — Adapted from the phylogeny at http://cichlidresearch.com/fish_html/cactinop.html (Nelson 2006). Tetraodontiformes (Fugu, Tetraodon) and Perciformes (cichlid, tilapia, sea bass, seabream, perch) are close phylogenetic relatives of Pleuronectiformes (turbot, halibut, sole). (PDF) [file pone.0075553.s001.pdf]

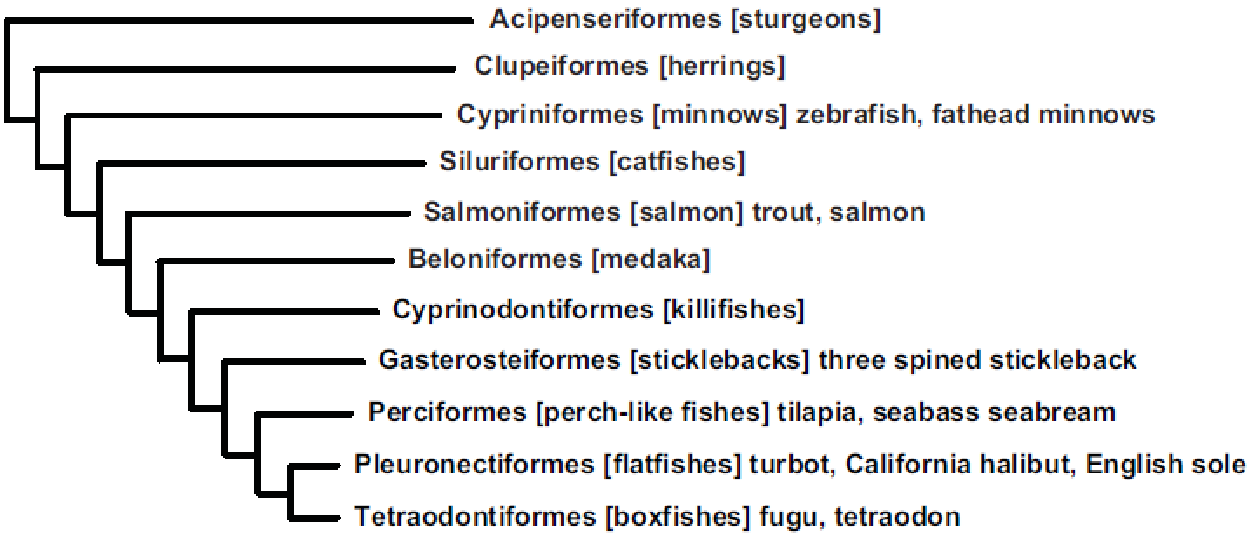

Figure S1
Flatfish (*Pleuronectiformes*) in an evolutionary context. Adapted from the phylogeny at http://cichlidresearch.com/fish_html/cactinop.html (Nelson 2006). Tetraodontiformes (Fugu, Tetraodon) and Perciformes (cichlid, tilapia, sea bass, seabream, perch) are close phylogenetic relatives of Pleuronectiformes (turbot, halibut, sole).
