# Supplementary material for: Molecular Analysis of Endocrine Disruption in Hornyhead Turbot at Wastewater Outfalls in Southern California Using a Second Generation Multi-Species Microarray
Source: PLoS One. 2013 Sep 25;8(9):e75553. doi: 10.1371/journal.pone.0075553 (PMC3783431; doi:10.1371/journal.pone.0075553)
Supplement: Figure S2 — (PDF) [file pone.0075553.s002.pdf]

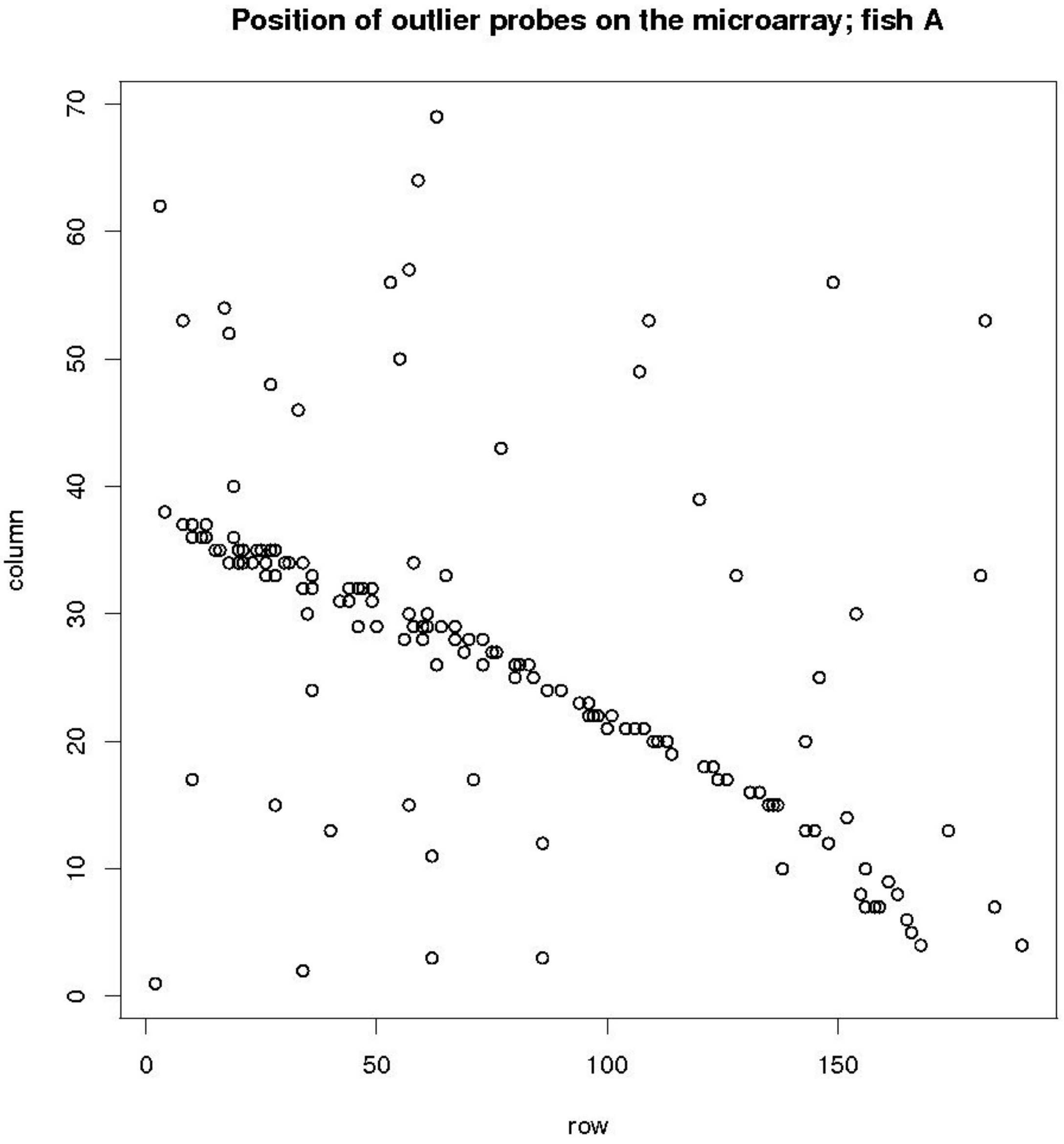

Figure S2
The probes were concentrated along a line, evidence that this represents a microarray artifact and that caution is warranted when performing differential expression measurements using microarrays.
