# Supplementary material for: Molecular Analysis of Endocrine Disruption in Hornyhead Turbot at Wastewater Outfalls in Southern California Using a Second Generation Multi-Species Microarray
Source: PLoS One. 2013 Sep 25;8(9):e75553. doi: 10.1371/journal.pone.0075553 (PMC3783431; doi:10.1371/journal.pone.0075553)
Supplement: Figure S3 — The X-axis represents microarray data (M) corresponding to three pooled control fish samples (A,B,C), the three individual control samples A, B, and C respectively and exposed fish sampled from sanitation districts in San Diego, Orange County, Dana Point, Los Angeles City and Los Angeles County. M specifically is a measure of differential gene expression (log2 (exposed /pooled control ABC) for all these samples. Only genes considered detected were depicted in these plots. The fish from impacted areas exhibited wider distributions in differentially expressed genes and outliers indicating that a large number of genes are differentially regulated with regard to control fish. Boxes represent the inter-quartile range, with the 75th percentile at the top and the 25th percentile at the bottom. The line in the middle of the box represents the 50th percentile, or median. Whiskers represent the rest of the distribution, with their terminations at 1.5 times the inter-quartile range in either direction. The individual notches represent outliers, i.e., unusually strongly modulated genes. (PDF) [file pone.0075553.s003.pdf]

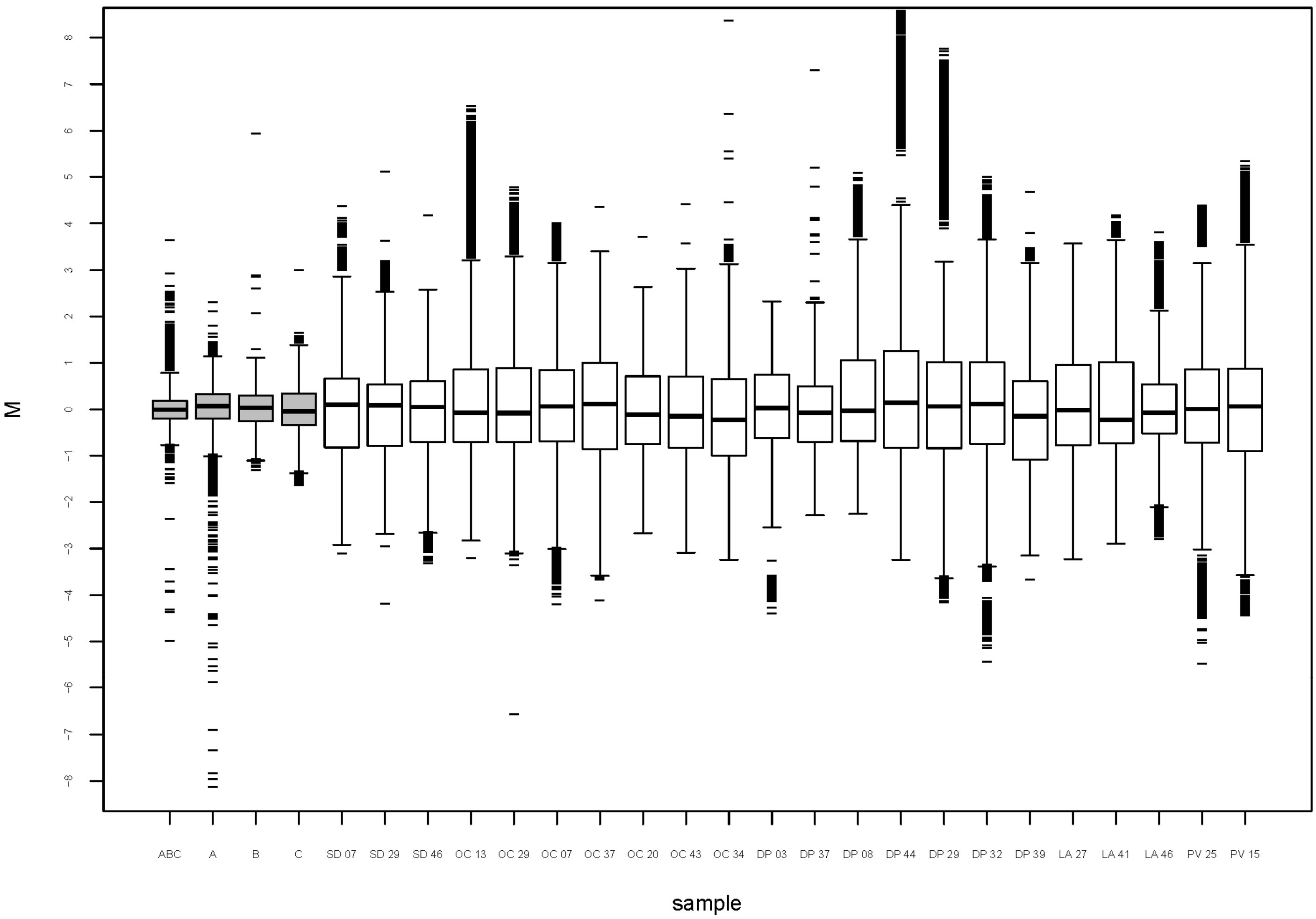

Figure S3

Examination of the feature intensity distribution in control and impacted fish livers.

The X-axis represents microarray data (M) corresponding to three pooled control fish samples (A,B,C), the three individual control samples A, B, and C respectively and exposed fish sampled from sanitation districts in San Diego, Orange County, Dana Point, Los Angeles City and Los Angeles County. M specifically is a measure of differential gene expression (log2 (exposed /pooled control ABC) for all these samples. Only genes considered detected were depicted in these plots. The fish from impacted areas exhibited wider distributions in differentially expressed genes and outliers indicating that a large number of genes are differentially regulated with regard to control fish. Boxes represent the inter-quartile range, with the 75th percentile at the top and the 25th percentile at the bottom. The line in the middle of the box represents the 50th percentile, or median. Whiskers represent the rest of the distribution, with their terminations at 1.5 times the inter-quartile range in either direction. The individual notches represent outliers, i.e., unusually strongly modulated genes.
