# Supplementary material for: Molecular Analysis of Endocrine Disruption in Hornyhead Turbot at Wastewater Outfalls in Southern California Using a Second Generation Multi-Species Microarray
Source: PLoS One. 2013 Sep 25;8(9):e75553. doi: 10.1371/journal.pone.0075553 (PMC3783431; doi:10.1371/journal.pone.0075553)
Supplement: Figure S4 — The fold changes were determined from log2 ratios between the probe signal of each fish and that of the pooled control sample. There were 326 unique sequence probes on the array, each of which was replicated in 46 distinct locations. The log2 ratio was calculated for each probe as the median of the 46 replicate log2 ratios. The 326 unique probes were subsequently sorted by their importance in descending order of the sum-squared statistic (i.e., sum of squares of log2 ratios across all fish). The top 100 probes were selected and included in this heat map. Since the majority of genes were represented on the microarray by sequences derived from several fish species, we further consolidated probes from the same gene, albeit different species, into a color strip of the same width. The range of colors is between -8-fold and +8-fold and preserves qualitative relationships among the individual values. All fold changes outside of this range have been truncated to ± 8. (PDF) [file pone.0075553.s004.pdf]

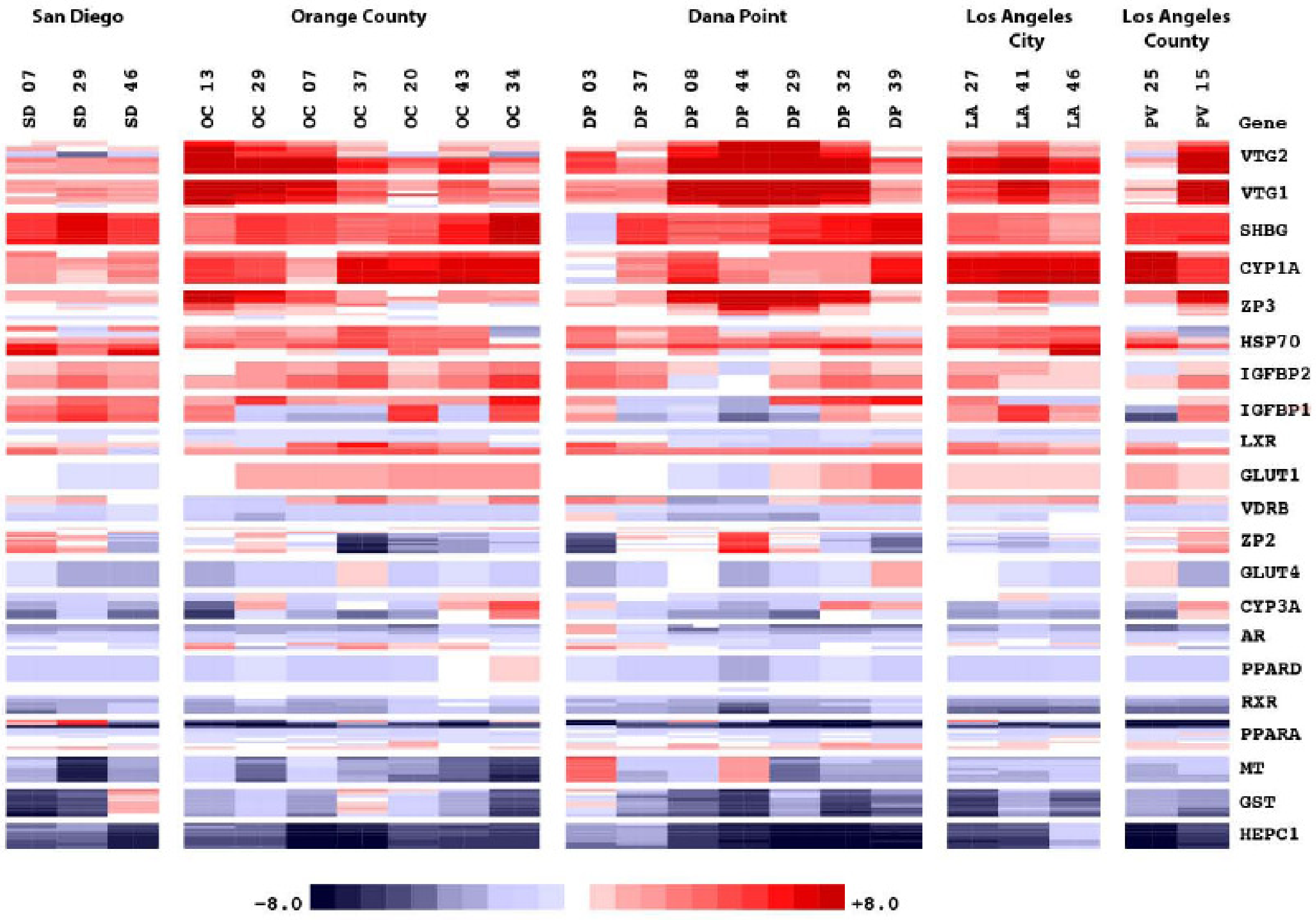

Figure S4
Feature level analysis of the top 100 differentially regulated multi-species probes.
The fold changes were determined from log2 ratios between the probe signal of each fish and that of the pooled control sample. There were 326 unique sequence probes on the array, each of which was replicated in 46 distinct locations. The log2 ratio was calculated for each probe as the median of the 46 replicate log2 ratios. The 326 unique probes were subsequently sorted by their importance in descending order of the sum-squared statistic (i.e., sum of squares of log2 ratios across all fish). The top 100 probes were selected and included in this heat map. Since the majority of genes were represented on the microarray by sequences derived from several fish species, we further consolidated probes from the same gene, albeit different species, into a color strip of the same width. The range of colors is between -8-fold and +8-fold and preserves qualitative relationships among the individual values. All fold changes outside of this range have been truncated to ± 8.
