# Supplementary material for: Molecular Analysis of Endocrine Disruption in Hornyhead Turbot at Wastewater Outfalls in Southern California Using a Second Generation Multi-Species Microarray
Source: PLoS One. 2013 Sep 25;8(9):e75553. doi: 10.1371/journal.pone.0075553 (PMC3783431; doi:10.1371/journal.pone.0075553)
Supplement: Table S1 — The gene and corresponding gene symbols are outlined. (PDF) [file pone.0075553.s005.pdf]

Gene	*Gene Symbol*
11beta-hydroxylase	*Cyp11b1*
17beta-hydroxysteroid dehydrogenase 2	*11 β-HSD2*
17beta-hydroxysteroid dehydrogenase type 1	*17β-HSD1*
Androgen Receptor	*AR*
Aromatase -cytochrome P450_19	*Cyp 19*
Aryl Hydrocarbon Receptor	*AhR*
Cytochrome P450 1A	*Cyp1A*
Cytochrome P450 3A	*Cyp3A*
Estrogen Receptor alpha	*ER α*
Estrogen Receptor beta	*ER β*
Farnesoid X receptor	*FXR*
Glucocorticoid Receptor	*GR*
Glutathione S-transferase alpha	*GST α*
Heat Shock Protein 70	*Hsp 70*
Heat Shock Protein 90A	*Hsp90 A*
Heat Shock Protein 90B	*Hsp90 B*
Hepcidin 1	*HEPC1*
Insulin-Like Growth Factor (IGF) Binding Protein 5	*IGFB*
Liver X Receptor	*LXR*
Metallothionein	*MT*
Mineralocorticoid receptor	*MR*
Peroxisome proliferator-activated receptor alpha	*PPAR α*
Peroxisome proliferator-activated receptor gamma	*PPAR γ*
Pregnane X Receptor	*PXR*
Progesterone Receptor	*PR*
Retinoic Acid Receptor	*RAR*
Retinoid X Receptor	*RXR*
Sex Hormone-binding Globulin	*SHBG*
Steroidogenic Acute Regulatory Protein	*STAR*
Thyroid Hormone Receptor alpha	*TR α*
Thyroid Hormone Receptor beta	*TR β*
Vascular Endothelial Growth Factor	*VEGF*
Vitamin D Receptor	*VDR*
Vitellogenin 1	*Vtg1*
Vitellogenin 2	*Vtg2*
Zona Pellucida Glycoprotein 2	*ZP2*
Zona Pellucida Glycoprotein 3	*ZP3*
28S Ribosomal RNA	*rRNA 28S*
Beta(cyto)-actin	*β-Actin*
Glyceraldehyde-3-Phosphate Dehydrogenase	*G3PDH*

Table S1

There are 37 diagnostic (endocrine) markers on the array. The gene and corresponding gene symbols are outlined.
