# Supplementary material for: Molecular Analysis of Endocrine Disruption in Hornyhead Turbot at Wastewater Outfalls in Southern California Using a Second Generation Multi-Species Microarray
Source: PLoS One. 2013 Sep 25;8(9):e75553. doi: 10.1371/journal.pone.0075553 (PMC3783431; doi:10.1371/journal.pone.0075553)
Supplement: Table S2 — The oligonucleotide probe sequences and the corresponding amplicon sizes are given. (PDF) [file pone.0075553.s006.pdf]

Gene	Transcript	Forward Primer 5' -> 3'	Reverse Primer 5' -> 3'	start	stop	amplicon (bp)
cyp4501A	*CYP1A*	TCACTGTGAGGACAGGAAGCT	CAGCACCGAACAGGTCATT	791	882	92
ERα	*ER* α	GGGTCACAATGACTATATGTGC	TTCATCATGCCCACTTCGTAAC	510	623	114
ERβ	*ER* β	ATCCGCCATGTCAGTAACAAA	GGAGCTGTGCATGATGTGG	1481	1597	117
VitA	*Vtg 1*	ATGAAGGGACAGACCTGTGG	AACCCAGGAATGAGCATAGC	502	618	117
VitB	*Vtg2*	ACTGGATGAGAGGCCAGACTT	GGTAGAACCCAGGAATGAGC	500	626	127

Table S2
SYBR green qPCR validation was carried out for *CYP1A, Vtg1, Vtg2, ER*α and *ER*β specific transcripts. The oligonucleotide probe sequences and the corresponding amplicon sizes are given.
